# Use of Surveillance Outbreak Response Management and Analysis System for Human Monkeypox Outbreak, Nigeria, 2017–2019

**DOI:** 10.3201/eid2602.191139

**Published:** 2020-02

**Authors:** Bernard C. Silenou, Daniel Tom-Aba, Olawunmi Adeoye, Chinedu C. Arinze, Ferdinand Oyiri, Anthony K. Suleman, Adesola Yinka-Ogunleye, Juliane Dörrbecker, Chikwe Ihekweazu, Gérard Krause

**Affiliations:** Helmholtz Centre for Infection Research, Braunschweig, Germany (B.C. Silenou, D. Tom-Aba, J. Dörrbecker, G. Krause);; PhD Programme “Epidemiology,” Braunschweig-Hannover, Germany (B.C. Silenou, D. Tom-Aba);; Nigeria Center for Disease Control, Abuja, Nigeria (O. Adeoye, C.C. Arinze, F. Oyiri, A.K. Suleman, A. Yinka-Ogunleye, C. Ihekweazu);; German Center for Infection Research, Braunschweig (G. Krause)

**Keywords:** monkeypox virus, digital health, mobile health, mHealth, outbreak detection, surveillance, Nigeria, Africa, contact tracing, viruses, Surveillance Outbreak Response Management and Analysis System, SORMAS

## Abstract

In November 2017, the mobile digital Surveillance Outbreak Response Management and Analysis System was deployed in 30 districts in Nigeria in response to an outbreak of monkeypox. Adaptation and activation of the system took 14 days, and its use improved timeliness, completeness, and overall capacity of the response.

Human monkeypox is a severe and rare smallpox-like illness that occurs sporadically in remote villages in the tropical rain forest of West and Central Africa (*1*,*2*). The causative agent, monkeypox virus, is transmitted by animal-to-human and human-to-human contact (*3*,*4*). In September 2017, an outbreak of monkeypox occurred in Nigeria after 40 years of no reported cases in the country. As of October 2017, local health departments in Nigeria had reported 89 cases and 294 contact persons (*5*,*6*). 

Early in the outbreak, the Nigeria Centre for Disease Control (NCDC) used a conventional surveillance system for the outbreak investigation. That system consisted of paper-based forms transferred manually to databases within the framework of the Integrated Disease Surveillance and Response System (*7*). As the outbreak expanded, NCDC faced challenges because of information delay and difficulties with updating and verifying case data, integrating laboratory tests, and managing contact tracing in the conventional system. In October 2017, NCDC decided to implement the Surveillance, Outbreak Response Management and Analysis System (SORMAS) on an ad hoc basis; an earlier prototype of this system had been successfully piloted in Nigeria in 2015 (*8*). SORMAS is an open-source mHealth (mobile health) system that organizes and facilitates infectious disease control and outbreak management procedures in addition to disease surveillance and epidemiologic analysis for all administrative levels of a public health system (*9*–*11*). SORMAS includes specific interfaces for 12 users (e.g., laboratorian, contact tracing officer, epidemiologist), disease-specific process modules for 12 epidemic-prone diseases, and a customizable process module for unforeseen emerging diseases; it adheres to the Integrated Disease Surveillance and Response System. Most users operate SORMAS on mobile digital devices (e.g., smartphone, tablet), bidirectionally synchronized with a central server via mobile telecommunication networks.

We compared SORMAS performance with that of the conventional surveillance system. Here we describe how we adapted and deployed SORMAS, discuss challenges encountered during implementation, and provide recommendations for deployment of similar mHealth tools.

## The Study 

In the second week of October 2017, we held a 2-day design thinking workshop with clinicians, epidemiologists, and virologists, in which all specific procedures for surveillance and response were defined in accordance with guidelines from the World Health Organization (*12*). Within 10 days, we translated the findings of the workshop into process models and programmed them into the existing SORMAS. A 2-day field test guided final programming revisions, which took another 2 days before the new module was released. In total, it took 14 days from initial decision to adapt and use SORMAS until its deployment. 

In November 2017, we trained the laboratory officers and district surveillance notification officers (DSNOs) in 30 of the most affected local government areas of 8 federal states (Appendix); each training session lasted 2 days. DSNOs used the mobile SORMAS version on mobile tablets to notify cases and conduct contact tracing; laboratories used either laptops or tablets to notify test results in SORMAS. We trained staff at the incident command center of the NCDC how to process and analyze data within SORMAS. The incident command center also transferred data into SORMAS received through the conventional system from local government areas not yet using SORMAS. The conventional system frequently involved recontacting DSNOs by phone to correct or update case reports. The dashboard and statistics module in SORMAS generated the epidemiologic indicators needed for weekly situation reports. We used the network package in R software for visualization and follow-up on chains of transmission (*13*). We conducted qualitative interviews with the NCDC incident managers of the monkeypox outbreak with regard to timeliness, usefulness, and workload of the conventional system compared with SORMAS. For quantitative evaluation, we used a set of core variables to compare the percentage of completeness in SORMAS versus that of the conventional system.

**Figure 1:**
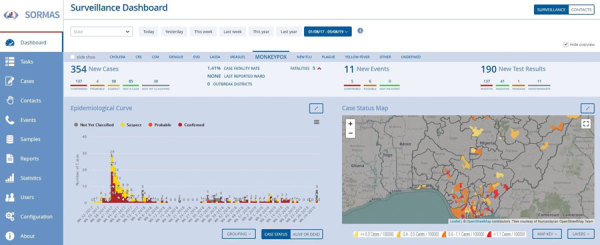
SORMAS dashboard showing monkeypox cases notified September 2017–July 2019 in Nigeria. The map shows the spatial spread of cases with local government area color by incidence proportion/100,000 population. The incidence proportion ranges from 0.1 (quartiles 0.3–0.7) to 8.1. During 2017, the number of cases by epidemic week increases gradually from week 32 to week 39, sharply increases in week 40, and gradually declines until week 53. Exportation of graphs, tables, and other epidemic indicators was generated in the statistic module of SORMAS. SORMAS, Surveillance Outbreak Response Management and Analysis System.

**Figure 2:**
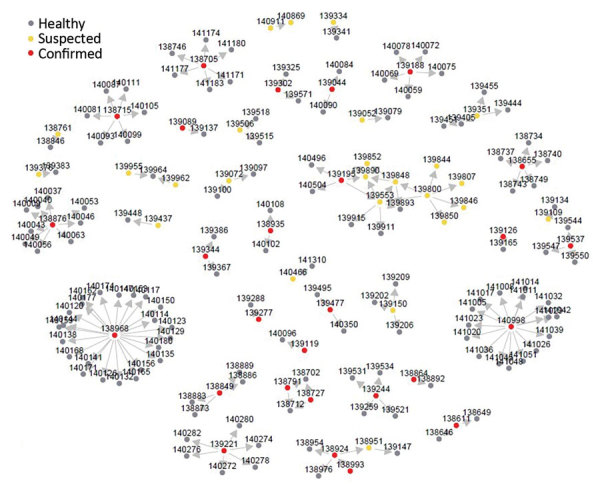
Network diagram for monkeypox cases and contact persons in Nigeria notified November 2017–July 2019. The nodes are labeled with unique identifiers for each person and colored by their classification status. Among case-patients, >1 contact person was reported for 57 (24%). The average number of contact persons/case-patient was 3 (quartiles 1–4, range 1–23). Arrows show the possible direction of infection transmission.

Yinka-Ogunleye et al. describe the epidemiologic characteristics of the outbreak in detail (*14*). From September 2017 through July 2019, including the period when SORMAS was not yet available, DSNOs reported 240 cases, either directly digitally in the field via SORMAS (n = 90) or via the conventional system (n = 150). Comparison of system attributes between SORMAS and the conventional system indicated equal or better performance of SORMAS for all attributes (Tables 1, 2). SORMAS continuously displayed the updated status of cases by case classification, epidemic curve, map of spatial distribution, contact persons, fatalities, and laboratory results, and it reported events in its dashboard within the incident command center (Figure 1). The dashboard also included performance indicators on contact tracing and case follow-up. The network diagrams linking case-patients to contact persons demonstrate that, of 167 contact persons, 12 (7%) converted to case-patients, of which 8 (66%) emerged from 1 chain of transmission (Figure 2).

**Table 1:** Qualitative comparison of attributes of SORMAS and the conventional surveillance system in response to monkeypox outbreak in Nigeria, November 2017–July 2019

Attribute	SORMAS	CS	Comments
Average time for data to arrive at NCDC from LGAs	2 min	2 d	For the CS, the DSNOs sent the paper case forms by post to NCDC, thus requiring longer time for case forms to arrive at NCDC.
Average time to update data (sample results from the laboratory, case classification, outcome, contacts) per case	5 min	20 min	Update in SORMAS requires searching for a case in the case directory and directly updating the fields. For the CS, the database was Excel (https://www.microsoft.com), and each type of case data was stored on a different Excel sheet, thus increasing the time and complexity of updating case data.
Workload to transfer cases from paper forms to database at NCDC	Less	More	With the CS, all case forms were entered in an Excel database at NCDC; with SORMAS, 90 (38%) of the 240 cases were entered directly from the field by DSNOs.
Availability of dashboard and statistics module to generate epidemiologic indicators for disease surveillance (e.g., case classification status, epidemic curve, laboratory test results, fatalities, and map of spatial distribution of cases and contact persons)	Yes	No	SORMAS had a dashboard that displayed the needed surveillance indicators; the CS did not.

*CS, conventional system; DSNOs, district surveillance notification officers; NCDC, Nigeria Centre for Disease Control; LGAs, local government areas; SORMAS, Surveillance Outbreak Response Management and Analysis System.

**Table 2:** Quantitative comparison of attributes of SORMAS and the conventional surveillance system in response to monkeypox outbreak in Nigeria, November 2017–July 2019*

Data availability for selected variables	SORMAS, %† n = 90	CS, %‡ n = 150	95% CI for difference
Sex	91	92	(−0.09 to 0.07)
Occupation	84	57	(0.15 to 0.39)
Date of birth	69	55	(0.00 to 0.27)
Onset date of symptoms	89	85	(−0.06 to 0.13)
Body temperature	53	3	(0.39 to 0.62)

*95% CI indicates difference in percentage of completeness determined by using 2-sample χ^2^ test. CS, conventional system; SORMAS, Surveillance Outbreak Response Management and Analysis System. †Percentage of completeness for monkeypox cases notified directly in SORMAS by district surveillance officers in the field.  ‡Percentage of completeness for monkeypox cases that arrived at the Nigeria Centre for Disease Control though the conventional system and were retrospectively registered in SORMAS.

## Conclusions

In this comparison, SORMAS clearly outperformed the conventional surveillance system. SORMAS accelerated visualization and analysis of case reports; expedited data updates and production of daily situation reports; and improved data completeness, timeliness, and several aspects of usefulness. The automated generation of chains of transmission enabled NCDC to assess overall transmissibility and effectiveness of contact tracing and helped with allocation of field staff during the outbreak.

The comparison of data completeness between SORMAS and the conventional system was limited by availability of data from the conventional system only after the incident command center had already executed data revisions and completions. Without this resource-intensive measure, the difference between SORMAS and the conventional system would have been more pronounced.

We also encountered challenges during the deployment phase. The ad hoc deployment of this new digital system in the midst of the outbreak allowed only 2 days of training for DSNOs to become acquainted with the tool. It also resulted in running 2 systems in parallel. Because the SORMAS concept integrates continuous surveillance and response management but has not yet been used routinely, its full potential could not come into play as the outbreak unrolled in this particular situation. Other challenges included the complaint of DSNOs not receiving compensation for transportation to execute follow-up visits for contact tracing, which could result in incomplete information about chains of transmission. This challenge, however, is not inherent to the conventional system or SORMAS, and SORMAS may have mitigated this challenge, given that it did produce chains of transmission that were not available by the conventional system.

Our evaluation was limited to selected attributes and based partly on quantitative analyses. Possibly the most convincing evidence for the added benefit of SORMAS was the ability of NCDC, while still responding to the monkeypox outbreak, to deploy SORMAS in 120 more local government areas of 6 federal states within 2 months. On the basis of the added value experienced through this measure, NCDC has set a goal to fully roll out SORMAS in all 774 local government areas of all 36 federal states plus the Federal Capital Territory in Nigeria by the end of 2021.

Overall, SORMAS has proven to be rapidly deployable and useful in response to multiple outbreaks, including an outbreak of an emerging disease such as monkeypox. For tools that integrate outbreak detection and response process management (such as SORMAS), we recommend their deployment independently from any response to an acute public health emergency to optimize efficiency of resources for software adaptation, hardware infrastructure, and training. Such a proactive approach will improve not only outbreak response but also early detection of outbreaks, thus further enhancing sustainability.

Appendix SORMAS deployment by federal states affected by monkeypox outbreak, Nigeria, October 30–November 15, 2017.
