# Supplementary material for: Use of Surveillance Outbreak Response Management and Analysis System for Human Monkeypox Outbreak, Nigeria, 2017–2019
Source: Emerg Infect Dis. 2020 Feb;26(2):345–9. doi: 10.3201/eid2602.191139 (PMC6986835; doi:10.3201/eid2602.191139)
Supplement: Appendix — SORMAS deployment by federal states affected by monkeypox outbreak, Nigeria, October 30–November 15, 2017. [file 19-1139-Techapp-s1.pdf]

Article DOI: https://doi.org/10.3201/eid2602.191139

Use of Surveillance Outbreak Response Management and Analysis System for Human Monkeypox Outbreak, Nigeria, 2017–2019

Appendix

Appendix Table. SORMAS deployment by federal states affected by monkeypox outbreak, Nigeria, October 30–November 15, 2017*

State	No. LGAs	No. LGAs affected†	No. SORMAS users‡
Bayelsa	8	2	19
Rivers	23	7	14
Cross-River	18	2	4
Delta	25	7	17
Akwa-Ibom	31	3	9
Lagos	20	3	10
Ekiti	16	3	9
Federal Capital Territory	6	3	7

*LGA, local government areas; SORMAS, Surveillance Outbreak Response Management and Analysis System.
†As at 2017;
‡District surveillance officers who were trained for 2 days each and issued a SORMAS tablet to notify cases and conduct contact tracing.
